# Evaluating the clinical utility of multimodal large language models in rare maculopathy

**DOI:** 10.1038/s41598-025-29299-2

**Published:** 2025-12-03

**Authors:** Melanie D. Tran, Evan Walker, Ines D. Nagel, Nehal Nailesh Mehta, Jesse Most, Henry A. Ferreyra, Lesley A. Everett, Paul Yang, Mark E. Pennesi, Shyamanga Borooah

**Affiliations:** 1 Retinal Division, Jacobs Retina Center, Shiley Eye Institute, University of California San Diego, 9415 Campus Point Dr, La Jolla, CA 92093 USA; 2 School of Medicine, University of California San Diego, La Jolla, CA 92037 USA; 3 Viterbi Family Department of Ophthalmology and Shiley Eye Institute, University of California San Diego, San Diego, CA USA; 4 Casey Eye Institute, Oregon Health and Science University, Portland, OR USA; 5 Retina Foundation of the Southwest, Dallas, TX USA

**Keywords:** Stargardt, Pentosan polysulfate sodium, Pattern dystrophy, Eye diseases, Hereditary eye disease

## Abstract

This study aimed to assess how multimodal large language models (MLLM) diagnose and differentiate Pentosan Polysulfate (PPS) Maculopathy from other phenotypic mimics. A retrospective review of clinical records and multimodal retinal imaging was conducted with patients from the Shiley Eye Institute and Casey Eye Institute. Four MLLMs (ChatGPT-4o, Claude 3.5 Sonnet, Google Gemini 1.5 Pro, Perplexity Llama 3.1 Sonar/Default) along with human retinal specialists answered prompts based on retinal imaging and demographic data. Performance was evaluated using accuracy, sensitivity and specificity estimates. The study included 126 eyes from 63 patients, with 36 eyes with PPS maculopathy, 50 eyes with Stargardt disease, and 40 eyes with *PRPH2*-associated multifocal pattern dystrophy. MLLMs showed improved accuracy and sensitivity when answer choices were restricted, with ChatGPT consistently performing best when all imaging modalities were prompted together. The inclusion of demographic data further enhanced performance in prompts with limited answer choices. Human retinal specialist evaluations aligned with MLLM performance trends and also improved with demographic data. While MLLMs show diagnostic potential, further refinement is needed before clinical implementation. These findings highlight the importance of prompt design and demographic data to optimize MLLM performance with retinal imaging modalities.

**Supplementary Information:**

The online version contains supplementary material available at 10.1038/s41598-025-29299-2.

## Introduction

Pentosan polysulfate sodium (PPS) is a synthetic glycosaminoglycan (GAG) like drug prescribed to treat the bladder pain syndrome interstitial cystitis (IC). PPS is the only FDA approved medication for IC^1^. A toxic retinopathy associated with PPS was first described in 2018^2^. PPS retinopathy results in pigmentary changes with altered autofluorescence and can be associated with vision loss resulting from atrophy, choroidal neovascularization, and cystoid macular edema. Studies have suggested a dose-dependent association for the prevalence of retinopathy with evidence of progression post-cessation^3,4^.

PPS maculopathy has often posed a diagnostic challenge as it shares phenotypic similarities with other retinal diseases such as Stargardt disease and pattern dystrophy. These include yellow deposits, altered autofluorescence, and retinal pigment epithelium (RPE) irregularities with atrophy in late-stage disease^5,6^. The literature includes previous reports describing how PPS maculopathy patients have been misdiagnosed with Stargardt disease or multifocal pattern dystrophy^7–10^. Stargardt disease is the most common inherited macular degeneration and is most commonly associated with pathogenic variants in the gene *ABCA4*^11,12^. Although traditionally associated with onset in childhood, an increasing number of late-stage adult Stargardt patients have been diagnosed with widescale genetic testing^5,6,13^. Multifocal pattern dystrophy, an inherited retinal disease associated with pathogenic variants in *PRPH2*, can present with similar features to Stargardt disease^14^. Given the differences in prognosis, management, and counseling for PPS Maculopathy, Stargardt disease, and multifocal pattern dystrophy, it is important to accurately identify differences between these diseases for general and retinal ophthalmologists.

There has been rapid progress in the use of artificial intelligence (AI) in retinal disease^15,16^. Recent studies have explored the application of AI via machine learning and deep learning in imaging in ophthalmology to diagnose age-related macular degeneration (AMD), diabetic retinopathy, glaucoma, papilledema, and retinopathy of prematurity (ROP)^17–22^. In 2022, OpenAI publicly released Chat Generative Pre-trained Transformer (ChatGPT), an AI-based large language model trained on large datasets which has the capability of accepting text input and responding with text output^23,24^. In 2023, ChatGPT-4 had a major upgrade which expanded the previous versions’ capabilities to include multimodal analysis including imaging^25^. Other so called multimodal large language models (MLLM), such as Claude, Gemini, and Perplexity, now feature similar capabilities. There have been limited studies evaluating the efficacy of AI and deep neural networks in imaging analysis, specifically for inherited retinal diseases such as retinitis pigmentosa^26,27^. While some studies have compared the ability of various MLLMs to respond to retinal and neuro-ophthalmology questions, comparative evaluations of their imaging analysis capabilities remain limited^28,29^. In our study, we compare the diagnostic accuracy of four different MLLMs to differentiate PPS Maculopathy, Stargardt Disease, and *PRPH2-*associated pattern dystrophy. To understand their utility in clinical practice, we compare their findings against those of retinal specialists.

## Methods

This is a retrospective cohort study. Patients who were seen by a retinal specialist at the University of California, San Diego (UCSD) Shiley Eye Institute between August 2019 to June 2024 or Oregon Health and Science University Casey Eye Institute for PPS Maculopathy, Stargardt Disease, or Pattern Dystrophy were selected for the study. Diagnosis of Stargardt Disease or Pattern Dystrophy was confirmed by genetic testing (Supplementary Tables S1 and S2). This study was approved by the UCSD Institutional Review Board (IRB #120516) and was granted a waiver of informed consent due to the retrospective nature of the work. No identifying patient information was included in the MLLM or human grading process including image metadata. The research was conducted according to the principles of the Declaration of Helsinki.

### Data and image collection

Demographic characteristics, including date of birth, age at date of imaging, gender, race, and ethnicity, were recorded for each patient. The following images were collected from Zeiss Forum Viewer (Version 4.2.4.15, Carl Zeiss, Oberkochen, Germany): ultrawidefield (UWF) pseudocolor fundus photography (UWF-C), UWF fundus autofluorescence (UWF-AF), and optical coherence tomography (OCT). UWF images were enlarged to the innermost borders for maximum image quality. Images were saved as .png files, anonymized, and contained no protected health information metadata. For each patient, images of both eyes were collected from the most recent visit days in which UWF-C, UWF-AF, and OCT imaging were all available.

### MLLM prompting

Prompts were developed to assess diagnostic accuracy in a stepwise manner to understand the effect of different imaging modalities and patient demographic information on diagnostic accuracy (Supplementary Table S3). All prompts were designed using recommendations for prompt engineering and prefaced with an opening statement “Answer the following prompt as an ophthalmologist.” to cue MLLMs to not assume any other background information unless given^30,31^. Prompt 1 assessed MLLM ability to diagnose imaging based on a multiple-choice list of common retinal diseases without any patient demographic information. Prompt 2 assessed each MLLM’s ability to integrate patient demographic information in diagnosis. Prompt 3 narrowed the scope of the answer choices to the diseases of interest in the study while excluding any patient demographic information. Prompt 4 again integrated patient demographic information. Each prompt consisted of multiple components designed to assess each MLLM’s ability to evaluate different imaging modalities both individually and in combination. In Prompts 1–4, modality A corresponded to the ultrawide-field color image, B to the ultrawide-field autofluorescence image, C to the OCT image, and D to all modalities combined. For each query, both eyes were presented simultaneously to better simulate clinical environments. MLLMs were then prompted to grade images where grading scales were available. Stargardt Disease severity was graded based on the Fishman Classification^32^. PPS Maculopathy severity was graded based on criteria previously established by Hanif et al.^33^

For each prompt, the date, time, diagnosis, and exact responses were recorded. The MLLMs evaluated in this study were ChatGPT-4o (OpenAI, San Francisco, CA, USA), Claude 3.5 Sonnet (Anthropic, San Francisco, CA, USA), Google Gemini 1.5 Pro (Google LLC, Mountain View, CA, USA), and Perplexity Llama 3.1 Sonar/Default (Perplexity AI, San Francisco, CA, USA). These models were selected because they represented the most advanced publicly available versions at the time of the study. The web-based interfaces of each model were chosen to better simulate clinical settings, where physicians may not be familiar with implementing APIs. To prevent ChatGPT-4o from training on previous inputs, a new chat with the “Temporary Chat” function activated was used for each set of images. In Perplexity Llama 3.1 Sonar/Default, “AI Data Retention” was disabled. No similar functions were available in Claude 3.5 Sonnet and Google Gemini 1.5 Pro. For each query, new chats were started to maintain independence from previous queries. Of note, different MLLMs had different requirements with regards to image uploads. ChatGPT-4o allowed for up to 10 images of size 20 MB or smaller per each query. Claude 3.5 Sonnet supported a maximum of 5 images of up to 10 MB each for each query. Google Gemini 1.5 Pro allowed for up to 10 images up to 7 MB per each image per each prompt. Lastly, Perplexity’s Llama 3.1 Sonar/Default allowed for up to 1 image per query. For prompts in which the required number of images would exceed the MLLM image upload capacity, the relevant images were collaged onto a single slide using Microsoft PowerPoint (version 16.89, Microsoft Corporation, Redmond, CA). Images were arranged on a white background to maximize their individual size without overlap, while maintaining borders to ensure separation without contact. No changes were otherwise made to the images, including but not limited to contrast, color, or brightness adjustment. Each slide was then saved as a .png file. Prompts and images were inputted into the MLLMs between August 2024 and January 2025.

### Human retinal specialist evaluation

All images were anonymized and randomized for each prompt during independent evaluation by human retinal specialists (HF, SB) (Supplementary Table S4). Human graders were asked to evaluate images with demographic information both with or without limited number of diagnoses to simulate clinical settings in which clinicians would have all patient information available. Ground truth for disease severity was based on grading by human retinal specialists in accordance with previously described criteria^32,33^. For eyes in which individual human grading differed, imaging was reviewed together with both retina specialists to establish a ground truth.

### Statistical analysis

For the summary of our study cohort, we present subject-level demographic characteristics, stratified by disease status. Cohort demographic characteristics are presented as count (%) and mean (95% CI) for categorical and continuous parameters, respectively. Interrater reliability of human retinal specialists’ evaluation of disease status was assessed using Cohen’s and Fleiss’s Kappa coefficients. Weighted Kappa coefficients were considered when evaluating the grading of ordinal disease severity. MLLM performance was evaluated per prompt using accuracy, sensitivity and specificity estimates. A clustered bootstrapping process was implemented at the subject-level with 3,000 resamples to produce 95% CI estimates of model performance metrics. Patient demographic and imaging data were collected once and processed once through each model–prompt combination to generate predictions. Clustered bootstrap resampling at the patient level was then used to quantify uncertainty. For each bootstrap iteration, a resampled dataset was drawn with replacement, performance metrics were recomputed, and bias-corrected 95% confidence intervals were derived from the empirical distributions. Between-model p-values were obtained from paired bootstrap distributions of metric differences. The bootstrap process was applied only to model outputs and did not involve re-feeding patient data to the MLLMs. The bootstrapping process was also utilized to compare performance between models. A Bonferroni correction was applied to adjust for multiple comparisons and control for family-wise error rate. All statistical analyses were conducted using the R programming language for statistical computation, version 4.4.0 (R Core Team [2024], R Foundation for Statistical Computing, Vienna, Austria.).

## Results

### Cohort summary

A total of 126 eyes from 63 patients were included in the study with 36 eyes from 18 PPS maculopathy patients, 50 eyes from 25 Stargardt patients, and 40 eyes from 20 *PRPH2-*associated multifocal pattern dystrophy. The average age at visit for PPS, Stargardt, and multifocal pattern dystrophy patients was 69.3 years, 40.4 years, and 53.1 years respectively (Supplementary Table S5). The sex distribution varied, with a higher proportion of females in the PPS Maculopathy group (72.2%) compared to pattern dystrophy (50.0%) and Stargardt Disease (48.0%) (Supplementary Table S5).

### MLLM performance

Due to the length of some of the prompts, these have not been listed in the text, but can be found in Supplementary Table S3.

### Disease diagnosis

#### Prompt 1

Prompt 1 assessed the ability of each MLLM to diagnose disease based solely on imaging either separately with UWF-C, UWF-AF, or OCT or all together, without patient demographic information. The MLLMs were provided with an inclusive list of differential diagnoses (Supplementary Table S3). When all three imaging modalities (UWF-C, UWF-AF, and OCT) were prompted together, MLLMs showed a low overall accuracy. ChatGPT demonstrated the highest overall accuracy [0.351 (0.25, 0.45)] and the highest mean sensitivity [0.267 (0.211, 0.319)]. By contrast, Claude showed the highest mean specificity [1 (1,1)] (Table 1). When only UWF-C images were prompted, Claude exhibited the greatest accuracy [0.125 (0.05, 0.204)] and mean sensitivity [0.147 (0.078, 0.215)], while Gemini demonstrated the highest mean specificity [0.992 (0.963, 1)]. For UWF-AF images, Gemini achieved the highest accuracy [0.387 (0.213, 0.532)] and mean sensitivity [0.372 (0.211, 0.51)], whereas Claude again showed the highest mean specificity [0.91 (0.859, 0.952)]. For OCT images, ChatGPT exhibited the highest accuracy [0.164 (0.081, 0.286)] and mean sensitivity [0.159 (0.076, 0.289)], while all four MLLMs displayed similar mean specificities (Table 1). Together, these results demonstrated that clinical diagnostic performance was poor when MLLMs were given many choices.

**Table 1:** MLLM performance summary of prompts 1 and 2.

Prompt	Model	Accuracy	Mean Sensitivity	Mean Specificity
1 A	ChatGPT	0.054 (0.017, 0.123)	0.042 (0.012, 0.093)	0.972 (0.933, 0.992)
	Claude	0.125 (0.05, 0.204)	0.147 (0.078, 0.215)	0.856 (0.796, 0.924)
	Gemini	0 (0, 0)	0 (0, 0)	0.992 (0.963, 1)
	Perplexity	0.054 (0.018, 0.103)	0.042 (0.014, 0.093)	0.981 (0.951, 1)
1B	ChatGPT	0.355 (0.238, 0.468)	0.298 (0.247, 0.397)	0.728 (0.687, 0.779)
	Claude	0.048 (0.016, 0.098)	0.048 (0.018, 0.097)	0.91 (0.859, 0.952)
	Gemini	0.387 (0.213, 0.532)	0.372 (0.211, 0.51)	0.714 (0.631, 0.781)
	Perplexity	0.339 (0.21, 0.419)	0.285 (0.204, 0.33)	0.699 (0.663, 0.745)
1 C	ChatGPT	0.164 (0.081, 0.286)	0.159 (0.076, 0.289)	0.958 (0.897, 0.99)
	Claude	0 (0, 0)	0 (0, 0)	0.992 (0.981, 1)
	Gemini	0.016 (0, 0.081)	0.019 (0, 0.076)	0.961 (0.927, 0.986)
	Perplexity	0.033 (0, 0.082)	0.027 (0, 0.07)	0.974 (0.934, 0.993)
1D	ChatGPT	0.351 (0.25, 0.45)	0.267 (0.211, 0.319)	0.709 (0.634, 0.761)
	Claude	0.036 (0, 0.096)	0.038 (0, 0.112)	1 (1, 1)
	Gemini	0.125 (0.038, 0.193)	0.097 (0.032, 0.148)	0.791 (0.735, 0.842)
	Perplexity	0.054 (0.017, 0.111)	0.056 (0.014, 0.136)	0.918 (0.865, 0.96)
2 A	ChatGPT	0.179 (0.111, 0.283)	0.144 (0.08, 0.259)	0.982 (0.946, 1)
	Claude	0.214 (0.123, 0.316)	0.21 (0.108, 0.339)	0.974 (0.946, 0.991)
	Gemini	0.018 (0, 0.069)	0.014 (0, 0.056)	1 (1, 1)
	Perplexity	0.179 (0.091, 0.25)	0.144 (0.09, 0.241)	0.974 (0.941, 0.992)
2B	ChatGPT	0.306 (0.175, 0.444)	0.272 (0.186, 0.38)	0.879 (0.803, 0.92)
	Claude	0.194 (0.1, 0.365)	0.169 (0.099, 0.308)	0.929 (0.891, 0.969)
	Gemini	0.355 (0.226, 0.452)	0.337 (0.218, 0.436)	0.926 (0.88, 0.948)
	Perplexity	0.262 (0.153, 0.383)	0.238 (0.149, 0.352)	0.873 (0.827, 0.938)
2 C	ChatGPT	0.148 (0.081, 0.262)	0.136 (0.075, 0.25)	0.961 (0.921, 0.986)
	Claude	0.066 (0.018, 0.127)	0.064 (0.027, 0.126)	1 (1, 1)
	Gemini	0.033 (0, 0.098)	0.032 (0.012, 0.087)	1 (1, 1)
	Perplexity	0.049 (0.016, 0.148)	0.04 (0.012, 0.107)	0.984 (0.965, 1)
2D	ChatGPT	0.298 (0.183, 0.404)	0.242 (0.155, 0.345)	0.89 (0.843, 0.954)
	Claude	0 (0, 0)	0 (0, 0)	0.984 (0.957, 1)
	Gemini	0 (0, 0)	0 (0, 0)	1 (1, 1)
	Perplexity	0.161 (0.093, 0.263)	0.14 (0.069, 0.242)	0.943 (0.9, 0.977)

#### Prompt 2

Prompt 2 assessed each MLLM’s ability to analyze images together with patient demographic information and to identify the diseases of interest from an inclusive list of differential diagnoses (Supplementary Table S3). UWF-C, UWF-AF, and OCT imaging was either provided individually or all together. When all three imaging modalities were prompted together, accuracy reduced from Prompt 1D to 2D in all MLLMs except for Perplexity (Table 1). ChatGPT again demonstrated the highest accuracy [0.298 (0.183, 0.404)] and mean sensitivity [0.242 (0.155, 0.345)], while the mean specificity across the MLLMs ranged between 0.89 and 1 (Table 1). When only UWF-C imaging was used, Claude exhibited the highest accuracy [0.214 (0.123, 0.316)] and mean sensitivity [0.21 (0.108, 0.339)]. For UWF-AF images, Gemini had the highest accuracy [0.355 (0.226, 0.452)] and mean sensitivity [0.337 (0.218, 0.436)]. As with Prompt 1, ChatGPT again had the highest accuracy [0.148 (0.081, 0.262)] and mean sensitivity [0.136 (0.075, 0.25)] when prompted with OCT images. For all three individual imaging modalities, the mean specificity across all MLLMs was consistent (Table 1). In summary, adding demographic data did not improve accuracy or sensitivity in all models, demonstrating the difficulty of diagnosing PPS maculopathy and its phenotypic mimetics in the setting of a broad list of differential diagnoses.

#### Prompt 3

After noting the poor overall performance of MLLMs in diagnosing disease correctly from an extended list of retinal conditions, Prompt 3 evaluated each MLLM’s ability to differentiate PPS maculopathy from Stargardt Disease and multifocal pattern dystrophy by narrowing down the available answer choices to these three diseases to see if performance improved (Supplementary Table S3). No patient demographic information was provided in this prompt. When all imaging modalities were prompted together, MLLM performance increased with the smaller number of choices. ChatGPT again achieved the highest accuracy [0.421 (0.298, 0.547)], while all four MLLMs demonstrated similar mean sensitivities and specificities (Table 2). For UWF-C images, ChatGPT was the most accurate [0.429 (0.281, 0.542)], Claude exhibited the highest mean sensitivity [0.381 (0.333, 0.462)], and Perplexity had the greatest mean specificity [0.911 (0.858, 0.955)]. For UWF-AF images, Perplexity demonstrated the highest mean sensitivity [0.363 (0.274, 0.481)] and mean specificity [0.685 (0.635, 0.737)], while both Perplexity and ChatGPT had the same accuracy [0.403 (0.283, 0.5); 0.403 (0.288, 0.492)]. Lastly, for OCT imaging, consistent with the previous prompts, ChatGPT again achieved the highest accuracy [0.393 (0.267, 0.508)]. These findings suggested that performance improved by limiting diagnostic choices.

**Table 2:** MLLM performance summary of prompts 3 and 4.

Prompt	Model	Accuracy	Mean Sensitivity	Mean Specificity
3 A	ChatGPT	0.429 (0.281, 0.542)	0.343 (0.28, 0.397)	0.67 (0.636, 0.694)
	Claude	0.357 (0.237, 0.473)	0.381 (0.333, 0.462)	0.685 (0.666, 0.714)
	Gemini	0.339 (0.245, 0.491)	0.278 (0.198, 0.353)	0.631 (0.582, 0.679)
	Perplexity	0.161 (0.089, 0.263)	0.175 (0.102, 0.283)	0.911 (0.858, 0.955)
3B	ChatGPT	0.403 (0.288, 0.492)	0.333 (0.333, 0.333)	0.667 (0.667, 0.667)
	Claude	0.29 (0.175, 0.367)	0.333 (0.333, 0.333)	0.667 (0.667, 0.667)
	Gemini	0.371 (0.246, 0.484)	0.334 (0.253, 0.456)	0.661 (0.615, 0.711)
	Perplexity	0.403 (0.283, 0.5)	0.363 (0.274, 0.481)	0.685 (0.635, 0.737)
3C	ChatGPT	0.393 (0.267, 0.508)	0.341 (0.248, 0.454)	0.671 (0.628, 0.733)
	Claude	0.311 (0.21, 0.371)	0.352 (0.292, 0.394)	0.674 (0.651, 0.692)
	Gemini	0.361 (0.283, 0.492)	0.356 (0.24, 0.455)	0.669 (0.623, 0.732)
	Perplexity	0.164 (0.063, 0.254)	0.18 (0.071, 0.263)	0.585 (0.537, 0.625)
3D	ChatGPT	0.421 (0.298, 0.547)	0.32 (0.295, 0.333)	0.66 (0.646, 0.673)
	Claude	0.304 (0.186, 0.389)	0.32 (0.254, 0.381)	0.66 (0.637, 0.688)
	Gemini	0.321 (0.226, 0.474)	0.27 (0.197, 0.378)	0.635 (0.597, 0.684)
	Perplexity	0.286 (0.153, 0.37)	0.287 (0.223, 0.357)	0.643 (0.613, 0.691)
4 A	ChatGPT	0.518 (0.389, 0.61)	0.482 (0.364, 0.559)	0.76 (0.704, 0.799)
	Claude	0.464 (0.356, 0.593)	0.457 (0.348, 0.562)	0.746 (0.691, 0.808)
	Gemini	0.375 (0.267, 0.481)	0.363 (0.288, 0.498)	0.706 (0.659, 0.776)
	Perplexity	0.607 (0.481, 0.74)	0.616 (0.509, 0.787)	0.81 (0.761, 0.877)
4B	ChatGPT	0.532 (0.435, 0.639)	0.536 (0.411, 0.594)	0.775 (0.719, 0.811)
	Claude	0.419 (0.333, 0.556)	0.437 (0.338, 0.585)	0.725 (0.675, 0.778)
	Gemini	0.548 (0.413, 0.698)	0.56 (0.439, 0.709)	0.785 (0.723, 0.855)
	Perplexity	0.541 (0.393, 0.633)	0.552 (0.426, 0.659)	0.778 (0.707, 0.832)
4C	ChatGPT	0.459 (0.29, 0.59)	0.451 (0.364, 0.602)	0.734 (0.7, 0.802)
	Claude	0.361 (0.213, 0.466)	0.35 (0.229, 0.531)	0.698 (0.609, 0.749)
	Gemini	0.459 (0.317, 0.548)	0.477 (0.394, 0.639)	0.744 (0.701, 0.804)
	Perplexity	0.492 (0.362, 0.627)	0.499 (0.388, 0.63)	0.76 (0.712, 0.845)
4D	ChatGPT	0.561 (0.469, 0.695)	0.531 (0.44, 0.65)	0.782 (0.737, 0.855)
	Claude	0.429 (0.316, 0.556)	0.42 (0.307, 0.555)	0.73 (0.669, 0.79)
	Gemini	0.446 (0.37, 0.596)	0.435 (0.343, 0.595)	0.742 (0.702, 0.793)
	Perplexity	0.518 (0.407, 0.642)	0.491 (0.387, 0.571)	0.767 (0.705, 0.811)

#### Prompt 4

Prompt 4 provided the MLLMs with patient demographic information while assessing their ability to diagnose and differentiate the diseases of interest in the study (Supplementary Table S3). When all images were prompted together, ChatGPT again demonstrated the best overall performance (Table 2). However, when considering individual imaging modalities, ChatGPT was outperformed by Perplexity for UWF-C and OCT images, and by Gemini for UWF-AF images (Table 2). In summary, the performance of MLLMs improved in terms of accuracy and sensitivity from Prompt 2D to Prompt 4D when they were provided with a limited set of potential diagnoses. Additionally, when all imaging modalities were prompted simultaneously, the best performance was observed with Prompt 4D, particularly when demographic information was included, and the answer choices were narrowed down.

### Human retinal specialist evaluation

Diagnosing and differentiating these conditions is often difficult even for experienced retinal providers. We next wanted to understand the context of MLLM performance by comparing against human retinal specialist grading. For human grading, Prompt 1 aligned with MLLM Prompt 2D, in which a comprehensive list of differential diagnoses was presented with patient demographic information (Supplementary Table S4). Human grading Prompt 2 was designed to match MLLM Prompt 4D in which graders were given only the diseases of interest along with patient demographic information. In Prompt 1, human graders had an agreement of 69.6% and an unweighted Cohen’s Kappa of 0.594 (*p* < 0.001). For Graders SB and HF, mean accuracy for Prompt 1 was far higher than that for MLLMs, with 0.714 (0.607, 0.804) and 0.571 (0.446, 0.643) respectively (Table 3). For Prompt 2, human graders had an increased agreement of 73.2% and an unweighted Cohen’s kappa of 0.592 (*p* < 0.001). Per diagnosis in Prompt 2, Fleiss’ kappa was 0.785 for PPS maculopathy, 0.563 for Stargardt Disease, and 0.420 for Pattern Dystrophy. For Prompt 2, mean accuracy was 0.839 (0.75, 0.893) and 0.804 (0.696, 0.893) respectively (Table 3). This was again far higher than compared with MLLMs. However overall, there were some disparities in answering even between experienced human graders.

**Table 3:** Human retinal specialist results summary.

Grader	Human Prompt ID	MLLM Prompt ID	Accuracy	Mean sensitivity	Mean specificity
SB	1	2D	0.714 (0.607, 0.804)	0.705 (0.566, 0.792)	0.921 (0.867, 0.959)
	2	4D	0.839 (0.75, 0.893)	0.822 (0.654, 0.906)	0.917 (0.865, 0.959)
HF	1	2D	0.571 (0.446, 0.643)	0.573 (0.468, 0.746)	0.914 (0.86, 0.948)
	2	4D	0.804 (0.696, 0.893)	0.808 (0.727, 0.959)	0.904 (0.856, 0.954)

### Disease staging

Human grader Prompt 3 focused on staging individual Stargardt eyes (Supplementary Table S4). In Prompt 3, human graders had an agreement or 64.6% and a Cohen’s kappa (squared distance weight) of 0.863 (*p* < 0.001). Individual accuracy in accordance with the ground truth ranged from 72.9% (56.2, 87.5) (HF) to 87.5% (68.8, 95.8) (SB) (Table 4). In Prompt 4, which graded individual PPS eyes, human graders had an agreement of 61.1% and a Cohen’s kappa (squared distance weight) of 0.327 (*p* < 0.001). Individual accuracy in Prompt 4 ranged between 72.2% (66.7, 66.7) (HF) to 88.9% (83.3, 94.4) (SB) (Table 4).

**Table 4:** Disease staging performance by retinal specialists and MLLMs.

Grader	Prompt	Accuracy (agreement %)	Kappa (unweighted)	Kappa (equal distance weight)
*Stargardt disease*				
SB	3	87.5 (68.8, 95.8)	0.825 (0.628, 0.969)	0.896 (0.754, 0.982)
HF	3	72.9 (56.2, 87.5)	0.633 (0.424, 0.851)	0.763 (0.58, 0.896)
ChatGPT	5B	12.5 (2.1, 22.9)	− 0.057 (− 0.168, 0.038)	− 0.173 (− 0.319, − 0.004)
Claude	5B	6.3 (2.1, 25.1)	− 0.065 (− 0.136, − 0.016)	− 0.038 (− 0.136, 0.053)
Gemini	5B	8.3 (4.2, 16.7)	0.000 (0.000, 0.000)	0.000 (0.000, 0.000)
Perplexity	5B	22.9 (8.3, 35.4)	0.066 (− 0.048, 0.162)	0.072 (− 0.019, 0.215)
*PPS maculopathy*				
SB	4	88.9 (83.3, 94.4)	0.812 (0.675, 0.906)	0.755 (0.566, 0.920)
HF	4	72.2 (66.7, 66.7)	0.547 (0.482, 0.482)	0.613 (0.386, 0.782)
ChatGPT	5B	30.6 (13.9, 47.2)	0.007 (− 0.166, 0.217)	0.031 (− 0.179, 0.209)
Claude	5B	27.8 (11.1, 41.7)	− 0.041 (− 0.310, 0.139)	− 0.077 (− 0.304, 0.102)
Gemini	5B	50.0 (25.0, 63.9)	0.020 (− 0.098, 0.189)	0.026 (− 0.091, 0.154)
Perplexity	5B	22.2 (8.3, 33.3)	− 0.079 (− 0.243, 0.075)	− 0.071 (− 0.273, 0.010)

Prompt 5 of the MLLM prompts focused on disease staging based on the ground truth established by human graders (Supplementary Table S3). Prompt 5A was designed to assess disease staging using the imaging modality most appropriate for diagnosing Stargardt Disease and PPS maculopathy, while Prompt 5B incorporated all imaging modalities. For disease staging, autofluorescence images were considered the most appropriate modality for Stargardt disease, whereas color fundus photographs were considered the most appropriate for PPS maculopathy. In Prompt 5B, Perplexity demonstrated the greatest accuracy in Stargardt Disease staging [22.9 (8.3, 35.4)], while Gemini had the highest accuracy in staging PPS eyes [50.0 (25.0, 63.9)] (Table 4). For PPS maculopathy disease staging, Gemini exhibited the lowest misclassification rate when MLLMs were prompted with both only UWF-C images and all imaging modalities (Table 5). When evaluating the severity of Stargardt Disease patients, ChatGPT had the lowest misclassification rate (77.1%) when MLLMs were given only UWF-AF images compared to Perplexity (77.1%) when all images were prompted together (Table 5).

**Table 5:** MLLM disease severity performance.

Disease	MLLM prompt	MLLM model	Accuracy (%)	Misclassified (%)
Stargardt disease	5A	ChatGPT	22.9	77.1
		Claude	12.5	87.5
		Gemini	14.6	85.4
		Perplexity	12.5	87.5
	5B	ChatGPT	12.5	87.5
		Claude	6.3	93.7
		Gemini	8.3	91.7
		Perplexity	22.9	77.1
PPS maculopathy	5A	ChatGPT	19.4	80.6
		Claude	11.1	88.9
		Gemini	41.7	58.3
		Perplexity	11.1	88.9
	5B	ChatGPT	30.6	69.4
		Claude	27.8	72.2
		Gemini	50.0	50.0
		Perplexity	22.2	77.8

Misclassification errors by MLLMs were further evaluated to identify trends in over and under classifications based on ground truth disease staging established by human graders. In Prompt 5B, Grade 1 PPS eyes were most frequently misclassified with the exception of Gemini which had a greater misclassification rate for Grade 3 PPS eyes (Table 6). For misclassified Grade 2 PPS eyes, MLLMs were more likely to over-classify rather than under classify in Prompt 5B with all imaging modalities (Table 6). A similar trend was observed in Prompt 5A in which MLLMs were asked to evaluate PPS eyes using only UWF-C images. All MLLMs, except for Gemini, were more likely to over classify Grade 2 PPS eyes in Prompt 5 A (Supplementary Table S6). For Stargardt’s disease, most MLLMs misclassified Stage 3 and 4 eyes as a lower stage, while Stage 1 and 2 eyes were more likely to be over-classified. These trends were consistent when MLLMs were provided with either only UWF-AF images or all imaging modalities (Table 6 and Supplementary Table S6). Overall, MLLM performance in disease classification varied with lower grades or stages more likely to be over-classified and higher grades or stages more frequently under-classified.

**Table 6:** MLLM disease classification performance in MLLM prompt 5B.

Disease stage	Misclassified (%)	Under-classification (%)	Over-classification (%)
*Prompt 5B-PPS maculopathy*			
*ChatGPT*			
1	75.0	0.0	100.0
2	68.42	15.38	84.62
3	60.0	100.0	0.0
*Claude*			
1	83.33	0.0	100.0
2	68.42	38.46	61.54
3	60.0	100.0	0.0
*Gemini*			
1	91.67	0.0	100.0
2	10.53	0.0	100.0
3	100.0	100.0	0.0
*Perplexity*			
1	100.0	0.0	100.0
2	68.42	0.0	100.0
3	60.0	100.0	0.0
*Prompt 5B-Stargardt disease*			
*ChatGPT*			
1	100.0	0.0	100.0
2	100.0	0.0	100.0
3	89.47	52.94	47.06
4	55.56	100.0	0.0
*Claude*			
1	100.0	0.0	100.0
2	25.0	0.0	100.0
3	100.0	100.0	0.0
4	100.0	100.0	0.0
*Gemini*			
1	100.0	0.0	100.0
2	0.0	–	–
3	100.0	100.0	0.0
4	100.0	100.0	0.0
*Perplexity*			
1	100.0	0.0	100.0
2	25.0	0.0	100.0
3	57.89	100.0	0.0
4	100.0	100.0	0.0

## Discussion

The aim of this study was to understand new MLLM image analysis performance in the context of disease which is difficult to differentiate even for retinal specialists and to assess whether current MLLM performance is clinically useful. Previous studies have explored the use of ChatGPT in screening for diabetic retinopathy (DR) risk and assessing fundus images to determine DR disease severity, but further optimization is required before ChatGPT can be implemented clinically^34,35^. Other studies exploring the application of deep neural networks in imaging analysis of inherited retinal diseases such as retinitis pigmentosa have also demonstrated high sensitivity but have also acknowledged the need for further development of these algorithms prior to clinical application^26,27^. To date, there have been no studies exploring the clinical application of MLLMs in evaluating PPS maculopathy, Stargardt Disease, and multifocal pattern dystrophy.

Because MLLMs, such as ChatGPT, are publicly available, they are more widely accessible in comparison to deep learning models that require customized algorithms and have the potential to improve accessibility to screenings in resource-limited settings. Previous studies have suggested that ChatGPT has the potential to optimize the workflow in clinics by providing diagnoses at a faster rate, thereby decreasing the workload of ophthalmologists to increase the available time to interface with patients and provide more individualized care^17,36^. As MLLMs continue to evolve and are trained on different datasets, diseases may potentially be detected earlier to prevent progression to advanced stages and ultimately improve patient outcomes.

The findings from this study demonstrate that MLLMs offer varying degrees of diagnostic accuracy which also varies by imaging modality and the inclusion of patient demographic information. Overall, MLLM performance did not show an improvement in diagnostic accuracy between Prompts 1D and 2D when patient demographics were provided. However, between Prompts 3D and 4D, there was an improvement in performance when the diagnostic choices were limited and demographic information was included. MLLMs performed better when fewer answer choices were presented, reflecting their potential for more accurate differentiation when the application scope is more limited. This pattern may be explained by the fact that rare diseases, like those in this study, are difficult for the models to differentiate in the presence of a wide range of differential diagnoses. A more extensive and diverse dataset may improve clinical performance, especially for rare diseases like PPS maculopathy, Stargardt disease, and multifocal pattern dystrophy given that these diseases can present with overlapping characteristics.

**Fig. 1:**
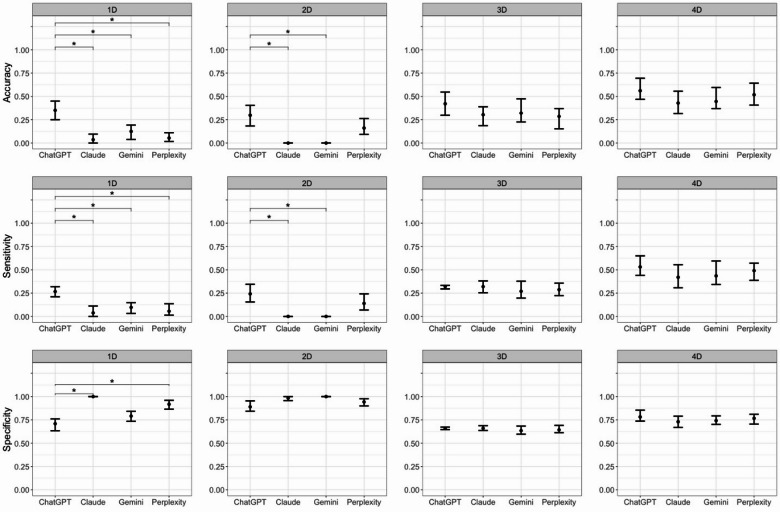
MLLM Comparison for Prompts 1D, 2D, 3D, and 4D.

Of interest were the prompts in which all three imaging modalities (UWF-C, UWF-AF, and OCT) were prompted together either with or without patient demographic information. These prompts were designed to better simulate clinical settings in which ophthalmologists would have all imaging available in addition to patient information. When all three imaging modalities were prompted together, ChatGPT consistently demonstrated the highest overall accuracy and sensitivity across prompts with noted statistically significant difference in accuracy, sensitivity and specificity in Prompts 1D and 2D (Fig. 1). This suggests that ChatGPT was particularly effective in integrating diverse imaging data. However, when imaging modalities were considered individually, other models outperformed ChatGPT in certain aspects. This may potentially allude to variation in available training datasets for different MLLMs suggesting a need for standardization of larger, high quality training sets prior to the integration of MLLMs into clinical settings. The inclusion of patient demographic information in Prompt 2 resulted in more nuanced performance across the MLLMs. While ChatGPT still maintained the highest accuracy, the mean sensitivity and specificity values across all MLLMs remained relatively consistent, with Claude performing well in in terms of accuracy for UWF-C and Gemini for UWF-AF. This suggests that the addition of demographics does not significantly shift the ability of these models to accurately diagnose conditions based solely on imaging but may improve the confidence in selecting the correct diagnosis. When looking at specifically Prompts 3D and 4D, which included a limited number of diagnoses and varied in the inclusion of demographic information, there were no statistically significant differences in accuracy, sensitivity, or specificity among MLLMs (Fig. 1). This again may allude to the difficulty of differentiating PPS maculopathy from other phenotypic mimics such as Stargardt Disease and multifocal pattern dystrophy without extensive training and additional medical history such as family history and medication history, which were both excluded from the prompts.

Human retinal specialists consistently outperformed MLLMs in diagnostic accuracy for Prompts 1 and 2, which corresponded to MLLM Prompts 2D and 4D, respectively. The higher agreement seen in Prompt 2 (73.2%) compared to Prompt 1 (69.6%), along with improved individual accuracy, likely reflects the reduced complexity of the task presented with fewer diagnostic options available (Table 3). One key observation from human grading was that accuracy improved when fewer diagnoses were included in the differential list. This finding was consistent across MLLM evaluations as well, highlighting the challenge posed by broad differential lists for both humans and MLLMs. For rare diseases, the presence of atypical or overlapping features can complicate accurate diagnosis. Of particular interest, retinal specialists noted that Stargardt patients in this study lacked retinal pigment epithelium (RPE) atrophy or diffuse flecks despite characteristic AF changes. Such discrepancies may account for the variability in grading accuracy and MLLM performance.

When focusing on disease staging, human graders demonstrated a wide range of accuracy, which may have varied depending on the severity and timing of disease progression in the images. These variations likely stem from the challenges of staging diseases that have atypical presentations or early-stage findings, which can be difficult to differentiate from other retinal conditions. In contrast, the MLLMs performed less accurately in disease staging. This performance discrepancy further underscores the complexity of retinal disease staging, where nuanced features such as the extent of RPE atrophy or fleck distribution play an essential role in staging, and MLLMs may lack sufficient training versus experienced retinal specialists.

The conclusions of the study in the context of utility of MLLMs in clinical practice may be limited by the manner in which data was presented to MLLMs. Although our study implemented best practices of prompt engineering, future investigations, ideally utilizing chain-of-thought prompting in static or version-controlled model environments, would provide further insight into strategies to optimize diagnostic performance. Nevertheless, we adopted a systematic stepwise approach to understand the effect of different imaging modalities and demographic data on diagnosis. In real-world clinical settings, additional contextual and longitudinal information may also influence diagnostic reasoning. Because demographic information was always provided to human retina specialists to better reflect real-world clinical practice, this design does not allow a direct comparison with MLLMs in contexts where only imaging is available such as at reading centers. Future studies could address this by evaluating imaging without demographic data. Although longitudinal changes were not evaluated in the current study, they also represent an important avenue for future research.

While MLLMs have the potential to be applied in clinical settings, there are other limitations in clinical practice. MLLMs, such as ChatGPT, are largely dependent on the available training datasets^37^. The process of building datasets may introduce an inherent bias based on patients who consent to the release of their medical records for training AI systems, and concerns around anonymization may limit the amount of available training data. During training, MLLMs are also vulnerable to external manipulation by selected inputs or to biases introduced by homogenous datasets^38^. Datasets may not include all potential presentations of disease, which may vary based on the demographics of the patients including age, gender, race, or ethnicity. For rare diseases, datasets for training may also not be readily available^39^. Due to a lack of access to external information, some MLLMs, such as ChatGPT, are continuously retrained to reflect new scientific findings and remain up-to-date^40–42^. In clinical practice, users must also be mindful of the phrasing of text inputs or prompts so as to not introduce bias or elicit biased answers.

Despite the highlighted challenges, the diagnostic capabilities of MLLMs are increasingly aligned with real-world clinical practice and human expert judgment, underscoring their potential utility in ophthalmology. However, transitioning from research settings to routine clinical use presents complex challenges, particularly regarding the generalizability of training datasets and the risk of bias that could exacerbate disparities in care for certain patient groups. In such cases, large-scale coordinated efforts across multiple institutions, coupled with rigorous clinical review of datasets prior to implementation, may facilitate the development of larger, more robust, and unbiased training sets in the future. Moreover, optimizing prompt engineering and incorporating comprehensive demographic and clinical data will be critical to address inherent limitations and ensure equitable application. Future studies should focus on incorporating genetic testing and additional clinical data to further enhance the diagnostic performance of MLLMs and to compare more ‘real world’ performance with human graders. With such advancements, MLLMs may be positioned to realize their full potential as transformative tools in ophthalmic care.

In conclusion, MLLMs offer great potential to aid clinical diagnosis and improve patient care. However, currently, in rare diseases such as the maculopathies studied in the present paper, MLLMs perform poorly when compared to retinal specialists, and their use is unlikely to aid clinical assessment. Future iterations of software with better training will likely enhance the performance of MLLMs. It remains to be seen when and how MLLMs will be implemented in clinical practice.

## Supplementary Information

Below is the link to the electronic supplementary material.

Supplementary Material 1

## Data Availability

All relevant data is listed in the manuscript or supplementary information files. Additional inquiries can be directed towards the corresponding author.

## Declarations

### Institution at which the study was conducted

University of California San Diego, La Jolla, CA.
