# Supplementary material for: Evaluating the clinical utility of multimodal large language models in rare maculopathy
Source: Sci Rep. 2025 Dec 3;16:54. doi: 10.1038/s41598-025-29299-2 (PMC12764543; doi:10.1038/s41598-025-29299-2)
Supplement: Supplementary file 1 — Supplementary Material 1 [file 41598_2025_29299_MOESM1_ESM.docx]

**Supplementary Table S1**. Genetic results of Stargardt patients

| **ID** | **Age at Visit** | **Gender** | **ABCA4 Variant** | **Heterozygosity** | **Pathogenicity** |
| --- | --- | --- | --- | --- | --- |
| 1S | 20 | F | c.5318C>T (p.Ala1773Val) | Heterozygous | Pathogenic |
|  |  |  | c.1237A>T (p.Asn413Tyr) | Heterozygous | Pathogenic |
| 5S | 16 | M | c.4577C>T (p.Thr1526Met) | Heterozygous | Pathogenic |
|  |  |  | c.5512C>Aj (p.His1838Asn) | Heterozygous | Pathogenic |
| 6S | 16 | F | c.5512C>T (p.His1838Tyr) | Heterozygous | Likely pathogenic |
|  |  |  | c.5324T>A (p.Ile1775Asn) | Heterozygous | Likely pathogenic |
| 8S | 64 | M | c.4253+43G>A | Heterozygous | Pathogenic |
|  |  |  | c.2588G>C (p.Gly863Ala) | Heterozygous | Pathogenic |
|  |  |  | c.2609C>T (p.Pro870Leu) | Heterozygous | Likely pathogenic |
|  |  |  | c.5603A>T (p.Asn1868Ile) | Heterozygous | Risk Factor |
| 11S | 69 | F | c.3292C>T (p.Arg1098Cys) | Heterozygous | Pathogenic |
|  |  |  | c.4139C>T (p.Pro1380Leu) | Heterozygous | Pathogenic |
| 15S | 39 | F | c.5898+1G>A (Splice donor) | Heterozygous | Pathogenic |
|  |  |  | c.5882G>A (p.Gly1961Glu) | Heterozygous | Pathogenic (low penetrance) |
| 22S | 8 | M | c.3G>T (p.Met1?) | Heterozygous | Pathogenic |
|  |  |  | c.6383A>G (p.His2128Arg) | Heterozygous | Pathogenic |
| 24S | 36 | F | c.5461-10T>C (Intronic) | Heterozygous | Pathogenic |
|  |  |  | c.5882G>A (p.Gly1961Glu) | Heterozygous | Pathogenic (low penetrance) |
| 26S | 76 | M | c.6089G>A (p.Arg2030Gln) | Heterozygous | Pathogenic |
|  |  |  | c.5603A>T (p.Asn1868Ile) | Heterozygous | Risk Factor |
| 27S | 73 | F | c.1804C>T (p.Arg602Trp) | Heterozygous | Pathogenic |
|  |  |  | c.3205A>G (p.Lys1069Glu) | Heterozygous | VUS |
| 28S | 24 | M | c.834del (p.Asp279Ilefs*21) | Homozygous | Pathogenic |
| 29S | 26 | F | c.3898C>T (p.Arg1300*) | Heterozygous | Pathogenic |
|  |  |  | c.6306C>A (p.Asp2102Glu) | Heterozygous | Pathogenic |
| 30S | 67 | F | c.6089G>A (p.Arg2030Gln) | Heterozygous | Pathogenic |
|  |  |  | c.4318T>G (p.Phe1440Val) | Heterozygous | Likely pathogenic |
| 31S | 9 | M | c.1957C>T (p.Arg653Cys) | Heterozygous | Pathogenic |
|  |  |  | c.3184C>A (p.Leu1062Ile) | Heterozygous | Likely pathogenic |
| 32S | 21 | M | c.868C>T (p.Arg290Trp) | Homozygous | Pathogenic |
| 33S | 68 | F | c.1819G>C (p.Gly607Arg) | Heterozygous | Pathogenic |
|  |  |  | c.6320G>A (p.Arg2107His) | Heterozygous | Pathogenic |
| 35S | 64 | F | c.4537dup (p.Gln1513Profs*42) | Homozygous | Pathogenic |
| 36S | 15 | M | c.1819G>C (p.Gly607Arg) | Heterozygous | Pathogenic |
|  |  |  | c.5882G>A (p.Gly1961Glu) | Heterozygous | Pathogenic (low penetrance) |
| 37S | 30 | M | c.5882G>A (p.Gly1961Glu) | Heterozygous | Pathogenic |
|  |  |  | c.4919G>A (p.Arg1640Gln) | Heterozygous | Pathogenic (low penetrance) |
| 40S | 32 | F | c.6112C>T (p.Arg2038Trp) | Heterozygous | Pathogenic |
|  |  |  | c.5882G>A (p.Gly1961Glu) | Heterozygous | Pathogenic (low penetrance) |
| 41S | 37 | F | c.4519G>A (p.Gly1508Arg) | Heterozygous | Pathogenic |
|  |  |  | c.5461-10T>C (Intronic) | Heterozygous | Pathogenic |
| 42S | 61 | M | c.3898C>T (p.Arg1300*) | Heterozygous | Pathogenic |
|  |  |  | c.4139C>T (p.Pro1380Leu) | Heterozygous | Pathogenic |
| 43S | 52 | M | c.5461-10T>C (Intronic) | Heterozygous | Pathogenic |
|  |  |  | c.6119G>A(p.Arg2040GIn) | Heterozygous | Pathogenic |
|  |  |  | c.5603A>T (p.Asn1868Ile) | Heterozygous | Benign (reportable variant) |
| 44S | 16 | M | c.4139C>T (p.Pro1380Leu) | Homozygous | Pathogenic |
| 45S | 70 | M | c.5461-10T>C | Heterozygous | Pathogenic |
|  |  |  | c.4253+12C>T | Heterozygous | VUS |

**Supplementary Table S2**. Genetic results of Pattern Dystrophy patients

| **ID** | **Age at Visit** | **PRPH2 Variant** | **Heterozygosity** | **Pathogenicity** |
| --- | --- | --- | --- | --- |
| 4P | 64 | c.828+3A>T | Heterozygous | Pathogenic |
| 5P | 68 | c.828+3A>T | Heterozygous | Pathogenic |
| 7P | 61 | c.828+3A>T | Heterozygous | Pathogenic |
| 14P | 33 | c.629C>G, p.(Pro210Arg) | Heterozygous | Pathogenic |
| 18P | 68 | c.620A>G (p.Asp207Gly) | Heterozygous | Likely pathogenic |
| 22P | 48 | c.828+3A>T | Heterozygous | Pathogenic |
| 23P | 60 | c.828+3A>T | Heterozygous | Pathogenic |
| 29P | 53 | c.629C>G p.P210R | Heterozygous | Pathogenic |
| 30P | 37 | c.828+3A>T | Heterozygous | Pathogenic |
| 38P | 58 | c.659G>C (p.Arg220Pro) | Heterozygous | Likely pathogenic |
| 41P | 48 | c.648_649del p.P216fs (c.648_649del (p.Ser217Leufs*83) ) | Heterozygous | Likely pathogenic |
| 48P | 56 | c.658C>T (p.Arg220Trp) | Heterozygous | Pathogenic |
| 49P | 54 | c.748T>C (p.Cys250Arg) | Heterozygous | Pathogenic |
| 56P | 49 | c.457A>G, p.(Lys153Glu) | Heterozygous | Pathogenic |
| 58P | 60 | c.828+3A>T | Heterozygous | Pathogenic |
| 61P | 57 | c.394del (p.Gln132Lysfs*7) | Heterozygous | Pathogenic |
|  |  | Gly131 del1ggC | Heterozygous | Possible disease-causing |
| 63P | 79 | c.136C>T (p.Arg46*) | Heterozygous | Pathogenic |
| 67P | 31 | c.136C>T (p.Arg46*) | Heterozygous | Pathogenic |
| 69P | 34 | c.422A>G (p.Tyr141Cys) | Heterozygous | Pathogenic |
| 71P | 55 | c.948del (p.Trp316*) | Heterozygous | Pathogenic |

**Supplementary Table S3.** MLLM Prompts

| **Prompt ID** | **Question** |
| --- | --- |
| **1A** | Answer the following prompt as an ophthalmologist. Assume you do not know any background information about the patient such as age, sex, or ethnicity. For the following **ultrawide field color image**, what is the single most likely diagnosis?  Age related macular degeneration  Central serious chorioretinopathy  Diabetic maculopathy  Degenerative myopic maculopathy  Hypertensive Retinopathy  Stargardt disease  Pattern Dystrophy  Best Disease  Epiretinal membrane  Pentosan polysulfate sodium maculopathy  Cystoid macular edema  Other pathology  Normal macula |
| **1B** | Answer the following prompt as an ophthalmologist. Assume you do not know any background information about the patient such as age, sex, or ethnicity. For the following **ultrawide field autofluorescence image**, what is the single most likely diagnosis?  Age related macular degeneration  Central serious chorioretinopathy  Diabetic maculopathy  Degenerative myopic maculopathy  Hypertensive Retinopathy  Stargardt disease  Pattern Dystrophy  Best Disease  Epiretinal membrane  Pentosan polysulfate sodium maculopathy  Cystoid macular edema  Other pathology  Normal macula |
| **1C** | Answer the following prompt as an ophthalmologist. Assume you do not know any background information about the patient such as age, sex, or ethnicity. For the following **optical coherence tomography (OCT) image**, what is the single most likely diagnosis?  Age related macular degeneration  Central serious chorioretinopathy  Diabetic maculopathy  Degenerative myopic maculopathy  Hypertensive Retinopathy  Stargardt disease  Pattern Dystrophy  Best Disease  Epiretinal membrane  Pentosan polysulfate sodium maculopathy  Cystoid macular edema  Other pathology  Normal macula |
| **1D** | Answer the following prompt as an ophthalmologist. Assume you do not know any background information about the patient such as age, sex, or ethnicity. For the following **set of color, autofluorescence, and OCT images**, what is the single most likely diagnosis?  Age related macular degeneration  Central serious chorioretinopathy  Diabetic maculopathy  Degenerative myopic maculopathy  Hypertensive Retinopathy  Stargardt disease  Pattern Dystrophy  Best Disease  Epiretinal membrane  Pentosan polysulfate sodium maculopathy  Cystoid macular edema  Other pathology  Normal macula |
| **2A** | Answer the following prompt as an ophthalmologist. Given the age and sex of the patient and the following ultrawide field color image, what is the single most likely diagnosis?  Age related macular degeneration  Central serious chorioretinopathy  Diabetic maculopathy  Degenerative myopic maculopathy  Hypertensive Retinopathy  Stargardt disease  Pattern Dystrophy  Best Disease  Epiretinal membrane  Pentosan polysulfate sodium maculopathy  Cystoid macular edema  Other pathology  Normal macula |
| **2B** | Answer the following prompt as an ophthalmologist. Given the age and sex of the patient and the following ultrawide field autofluorescence image, what is the single most likely diagnosis?  Age related macular degeneration  Central serious chorioretinopathy  Diabetic maculopathy  Degenerative myopic maculopathy  Hypertensive Retinopathy  Stargardt disease  Pattern Dystrophy  Best Disease  Epiretinal membrane  Pentosan polysulfate sodium maculopathy  Cystoid macular edema  Other pathology  Normal macula |
| **2C** | Answer the following prompt as an ophthalmologist. Given the age and sex of the patient and the following optical coherence tomography (OCT) image, what is the single most likely diagnosis?  Age related macular degeneration  Central serious chorioretinopathy  Diabetic maculopathy  Degenerative myopic maculopathy  Hypertensive Retinopathy  Stargardt disease  Pattern Dystrophy  Best Disease  Epiretinal membrane  Pentosan polysulfate sodium maculopathy  Cystoid macular edema  Other pathology  Normal macula |
| **2D** | Answer the following prompt as an ophthalmologist. Given the age and sex of the patient and the following set of color, autofluoresence, and OCT images, what is the single most likely diagnosis?  Age related macular degeneration  Central serious chorioretinopathy  Diabetic maculopathy  Degenerative myopic maculopathy  Hypertensive Retinopathy  Stargardt disease  Pattern Dystrophy  Best Disease  Epiretinal membrane  Pentosan polysulfate sodium maculopathy  Cystoid macular edema  Other pathology  Normal macula |
| **3A** | Answer the following prompt as an ophthalmologist. Assume you do not know any background information about the patient such as age, sex, or ethnicity. For the following ultrawide field color image, what is the single most likely diagnosis?  Stargardt disease  Pattern Dystrophy  Pentosan polysulfate sodium maculopathy |
| **3B** | Answer the following prompt as an ophthalmologist. Assume you do not know any background information about the patient such as age, sex, or ethnicity. For the following ultrawide field autofluorescence image, what is the single most likely diagnosis?  Stargardt disease  Pattern Dystrophy  Pentosan polysulfate sodium maculopathy |
| **3C** | Answer the following prompt as an ophthalmologist. Assume you do not know any background information about the patient such as age, sex, or ethnicity. For the following optical coherence tomography (OCT) image, what is the single most likely diagnosis?  Stargardt disease  Pattern Dystrophy  Pentosan polysulfate sodium maculopathy |
| **3D** | Answer the following prompt as an ophthalmologist. Assume you do not know any background information about the patient such as age, sex, or ethnicity. For the following set of images, what is the single most likely diagnosis?    Stargardt disease  Pattern Dystrophy  Pentosan polysulfate sodium maculopathy |
| **4A** | Answer the following prompt as an ophthalmologist. Given the age and sex of the patient and the following ultrawide field color image, what is the single most likely diagnosis?  Stargardt disease  Pattern Dystrophy  Pentosan polysulfate sodium maculopathy |
| **4B** | Answer the following prompt as an ophthalmologist. Given the age and sex of the patient and the following ultrawide field autofluorescence image, what is the single most likely diagnosis?  Stargardt disease  Pattern Dystrophy  Pentosan polysulfate sodium maculopathy |
| **4C** | Answer the following prompt as an ophthalmologist. Given the age and sex of the patient and the following optical coherence tomography (OCT) image, what is the single most likely diagnosis?  Stargardt disease  Pattern Dystrophy  Pentosan polysulfate sodium maculopathy |
| **4D** | Answer the following prompt as an ophthalmologist. Given the age and sex of the patient and the following set of color, autofluoresence, and OCT images, what is the single most likely diagnosis?  Stargardt disease  Pattern Dystrophy  Pentosan polysulfate sodium maculopathy |
| **5Ai** | Answer the following prompt as an ophthalmologist. Assume each patient has Stargardt Disease. For the following ultrawide field autofluorescence image, what is the single most likely stage of disease based on the Fishman classification published in Fishman GA. Fundus flavimaculatus. A clinical classification. Archives of Ophthalmology. 1976;94(12):2061-2067 using the reference set of images and classification guide given below?  Stage 1: Features pigmentary changes in the macula and pisciform flecks within 1 disc diameter (DD) of the fovea.  Stage 2: Pisciform flecks are present beyond 1 DD from the margin of the fovea, extending beyond the arcades and nasally to the optic disc.  Stage 3: Diffuse resorption of flecks and choriocapillaris atrophy in the macula.  Stage 4: Diffuse resorption of flecks and extensive choriocapillaris/RPE atrophy throughout the fundus. |
| **5Aii** | Answer the following prompt as an ophthalmologist. Assume each patient has Pentosan Polysulfate Sodium Maculopathy. For the following ultrawide field color image, what is the single most likely stage of disease based on the Hanif classification?  Grade 1: The disease is contained within the vascular arcades without atrophy.  Grade 2: The disease extends to the temporal vascular arcades but does not span more than 2 disc diameters beyond the arcades, with or without noncentral atrophy.  Grade 3: The disease extends at least 2 disc diameters beyond the temporal vascular arcades and/or involves the presence of atrophy at the foveal center. |
| **5Bi** | Answer the following prompt as an ophthalmologist. Assume that this patient has Stargardt disease. Based on the given set of images and the Fishman classification, what is the single most likely diagnosis?  Stage 1: Features pigmentary changes in the macula and pisciform flecks within 1 disc diameter (DD) of the fovea.  Stage 2: Pisciform flecks are present beyond 1 DD from the margin of the fovea, extending beyond the arcades and nasally to the optic disc.  Stage 3: Diffuse resorption of flecks and choriocapillaris atrophy in the macula.  Stage 4: Diffuse resorption of flecks and extensive choriocapillaris/RPE atrophy throughout the fundus. |
| **5Bii** | Answer the following prompt as an ophthalmologist. Assume that this patient has Pentosan Polysulfate Maculopathy (PPS). Based on the given set of images and the grading criteria as described by Hanif et al, what is the single most likely grade?  Grade 1: The disease is contained within the vascular arcades without atrophy.  Grade 2: The disease extends to the temporal vascular arcades but does not span more than 2 disc diameters beyond the arcades, with or without noncentral atrophy.  Grade 3: The disease extends at least 2 disc diameters beyond the temporal vascular arcades and/or involves the presence of atrophy at the foveal center. |

**Supplementary Table S4.** Human retinal specialist prompts

| **Prompt ID** | **Question** |
| --- | --- |
| **1** | Answer the following prompt as an ophthalmologist. Based on the given set of images (color, autofluorescence and OCT), age, and sex, what is the single most likely diagnosis for each patient?  Age related macular degeneration (AMD)  Central serious chorioretinopathy (CSR)  Diabetic maculopathy (DR)  Degenerative myopic maculopathy  Hypertensive Retinopathy (HR)  Stargardt disease (SD)  Pattern Dystrophy (PD)  Best Disease  Epiretinal membrane (ERM)  Pentosan polysulfate sodium maculopathy (PPS)  Cystoid macular edema (CME)  Other pathology (OP)  Normal macula (NM) |
| **2** | Answer the following prompt as an ophthalmologist. Based on the given set of images (color, autofluorescence and OCT), age, and sex, what is the single most likely diagnosis?  Stargardt Disease (SD)  Pattern Dystrophy (PD)  Pentosan Polysulfate Maculopathy (PPS) |
| **3** | Answer the following prompt as an ophthalmologist. Assume that this patient has Stargardt disease. Based on the given set of images (color, autofluorescence and OCT) and the Fishman classification, what is the single most likely stage?  Stage 1: Features pigmentary changes in the macula and pisciform flecks within 1 disc diameter (DD) of the fovea.  Stage 2: Pisciform flecks are present beyond 1 DD from the margin of the fovea, extending beyond the arcades and nasally to the optic disc.  Stage 3: Diffuse resorption of flecks and choriocapillaris atrophy in the macula.  Stage 4: Diffuse resorption of flecks and extensive choriocapillaris/RPE atrophy throughout the fundus. |
| **4** | Answer the following prompt as an ophthalmologist. Assume that this patient has Pentosan Polysulfate Maculopathy (PPS). Based on the given set of images (color, autofluorescence and OCT) and the grading criteria as described by Hanif et al, what is the single most likely grade?  Grade 1: The disease is contained within the vascular arcades without atrophy.  Grade 2: The disease extends to the temporal vascular arcades but does not span more than 2 disc diameters beyond the arcades, with or without noncentral atrophy.  Grade 3: The disease extends at least 2 disc diameters beyond the temporal vascular arcades and/or involves the presence of atrophy at the foveal center. |

**Supplementary Table S5.** Demographic characteristics stratified by diagnosis

|  | Pattern Dystrophy  (n = 20 subjects) | PPS Maculopathy  (n = 18 subjects) | Stargardt  (n = 25 subjects) |
| --- | --- | --- | --- |
| **Age at Visit, years** | 53.1 (47.4, 58.8) | 69.3 (63.7, 74.9) | 40.4 (30.7, 50.0) |
| **Sex** |  |  |  |
| Female | 10 (50.0%) | 13 (72.2%) | 12 (48.0%) |
| Male | 10 (50.0%) | 5 (27.8%) | 13 (52.0%) |
| **Race** |  |  |  |
| Asian | 0 (0.0%) | 0 (0.0%) | 1 (4.0%) |
| Black or African American | 1 (5.0%) | 1 (5.6%) | 1 (4.0%) |
| Other Race or Mixed Race | 1 (5.0%) | 2 (11.1%) | 9 (36.0%) |
| Unknown or Not Reported | 16 (80.0%) | 0 (0.0%) | 4 (16.0%) |
| White | 2 (10.0%) | 15 (83.3%) | 10 (40.0%) |
| **Ethnicity** |  |  |  |
| Not Hispanic, Latino(a), or Spanish origin | 5 (25.0%) | 16 (88.9%) | 16 (64.0%) |
| Other Hispanic, Latino(a) or Spanish Origin | 0 (0.0%) | 1 (5.6%) | 4 (16.0%) |
| Other Pacific Islander | 0 (0.0%) | 0 (0.0%) | 1 (4.0%) |
| Unknown or Not Reported | 15 (75.0%) | 1 (5.6%) | 4 (16.0%) |

**Supplementary Table S6**. MLLM Disease Classification Performance in MLLM Prompt 5A

| **Prompt 5A - PPS Maculopathy** | | | |
| --- | --- | --- | --- |
| **Disease Stage** | **Misclassified (%)** | **Under-Classification (%)** | **Over-Classification (%)** |
| ChatGPT |  |  |  |
| 1 | 100.0 | 0.0 | 100.0 |
| 2 | 84.21 | 6.25 | 93.75 |
| 3 | 20.0 | 100.0 | 0.0 |
| Claude |  |  |  |
| 1 | 91.67 | 0.0 | 100.0 |
| 2 | 100.0 | 0.0 | 100.0 |
| 3 | 40.0 | 100.0 | 0.0 |
| Gemini |  |  |  |
| 1 | 33.33 | 0.0 | 100.0 |
| 2 | 63.16 | 83.33 | 16.67 |
| 3 | 100.0 | 100.0 | 0.0 |
| Perplexity |  |  |  |
| 1 | 100.0 | 0.0 | 100.0 |
| 2 | 94.74 | 0.0 | 100.0 |
| 3 | 40.0 | 100.0 | 0.0 |
| **Prompt 5A – Stargardt Disease** | | | |
| ChatGPT |  |  |  |
| 1 | 100.0 | 0.0 | 100.0 |
| 2 | 100.0 | 0.0 | 100.0 |
| 3 | 78.95 | 40.0 | 60.0 |
| 4 | 22.22 | 100.0 | 0.0 |
| Claude |  |  |  |
| 1 | 100.0 | 0.0 | 100.0 |
| 2 | 0.0 | - | - |
| 3 | 89.47 | 94.12 | 5.88 |
| 4 | 100.0 | 100.0 | 0.0 |
| Gemini |  |  |  |
| 1 | 81.25 | 0.0 | 100.0 |
| 2 | 0.0 | - | - |
| 3 | 100.0 | 100.0 | 0.0 |
| 4 | 100.0 | 100.0 | 0.0 |
| Perplexity |  |  |  |
| 1 | 100.0 | 0.0 | 100.0 |
| 2 | 0.0 | - | - |
| 3 | 94.74 | 88.89 | 11.11 |
| 4 | 88.89 | 100.0 | 0.0 |
